# Expression of LGI1 Impairs Proliferation and Survival of HeLa Cells

**DOI:** 10.1155/2009/417197

**Published:** 2009-10-07

**Authors:** Nadia Gabellini, Valentina Masola

**Affiliations:** Department of Biological Chemistry, University of Padova, 35121 Padova, Italy

## Abstract

The LGI1 gene was suggested to function as tumor suppressor for its ability to reduce malignant features of glioblastoma cells. In support to this proposal were the findings that overexpression of LGI1 in neuroblastoma cells inhibited proliferation and induced apoptosis. In this study we performed stable LGI1 expression in HeLa cells to examine whether the noxious effect of LGI1 might be extended to cancer cells of diverse origin. HeLa cell clones stably expressing LGI1 exhibited a significant impairment of proliferation and a consistent increase of cell death when compared with control cells lacking expression of LGI1. Expression of LGI1 increased the activity of apoptosis effectors caspase-3/7; furthermore it downregulated the antiapoptotic BCL2 gene and upregulated the proapoptotic BAX gene expression, suggesting that the cause of HeLa cells death might be an increased susceptibility to apoptosis induced by LGI1. The results suggested that LGI1 is capable to restrain growth and survival of adenocarcinoma cells such as HeLa.

## 1. Introduction

The leucine-rich, glioma inactivated 1 (LGI1) gene was identified at the brake point of a reciprocal chromosome translocation t(10;19)(q24;q13) in the glioblastoma cell line T98G [1]. LGI1 protein includes domains presumably involved in protein-protein interaction such as the leucine-rich repeats flanked by cysteine rich regions and the seven-bladed beta-propeller domain [2–4]. LGI1 gene is predominantly expressed in brain, and it is associated with autosomal dominant lateral temporal epilepsy (ADLTE) [5, 6]. Although the specific function of LGI1 remains to be defined, it was suggested to form complexes with Kv1.1 potassium channels, essential regulators of synaptic transmission in brain and also to be a ligand of the trans-membrane receptor ADAM22, a protein highly expressed in brain whose function is still uncertain [7, 8].

The loss of LGI1 expression in most high-grade gliomas supported the function of tumor suppressor gene [1]. In agreement with this hypothesis the reexpression of LGI1 in glioma cells, lacking endogenous LGI1, impaired cell growth and migration capacity [9]. These phenotypic changes were linked with the downregulation of matrix metalloproteinase (MMP) genes by LGI1-mediated inhibition of the ERK/MAPK pathway [10]. A role of LGI1 in the development of malignant brain tumors was substantiated by the discovery of somatic missense mutations in LGI1 gene associated with high- and low-grade gliomas [11]. The ability of LGI1 to suppressor growth of neuroblastoma cells was suggested by the results of LGI1 overexpression, which was shown to impair proliferation and to induce apoptosis through the inhibition of the PI3K/AKT pathway [12, 13]. Further support to the tumor suppressor function of LGI1 was provided by a comprehensive study on malignant esophageal tumors (Barrett's-related adenocarcinomas) showing that expression of LGI1 was consistently downregulated [14].

The aim of this study was to establish whether LGI1 was capable to suppress growth of cancer cells different from glioblastoma and neuroblastoma. For this purpose we performed stable expression of LGI1 in HeLa cell, which is derived from a human cervical adenocarcinoma and expresses the human papillomavirus proteins E6/E7 (HPV-18) [15]. Essentially these viral proteins sustain cell proliferation and survival through the inactivation of tumor suppressors p53 and pRB, which are implicated in cell cycle arrest and induction of apoptosis [16, 17]. For these features conferring remarkably vigorous growth, HeLa cells appeared to be especially appropriate to test the effects of LGI1.

The results showed that expression of LGI1 inhibited the rate of HeLa cell proliferation and increased cellular death. Furthermore LGI1 altered expression of key regulators of apoptosis such as the antiapoptotic B-cell lymphoma 2 gene (BCL2) and of the proapoptotic B-cell lymphoma 2-associated X protein gene (BAX) [18]. The lethal effect produced by LGI1 expression in HeLa cells suggests that the proposed role of tumor suppressor might be extended to adenocarcinoma-derived cells.

## 2. Materials and Methods

### 2.1. Cell Culture and Transfection

HeLa cells (ATCC Number: CCL-2) were cultured in Dulbecco's Modified Eagle medium (DMEM, low glucose) with 10% heat inactivated Fetal Calf Serum (FCS). For transfection cells (1.5 × 10^6^) were seeded on 10 cm diameter plastic dishes. Transfection with plasmid pcLGI1 including the human LGI1 cDNA (2075 bp) or with empty vector pcDNA3 (10 *μ*g/mL each; Invitrogen, Groningen, The Netherlands) was performed by the DNA Ca^2+^-phosphate coprecipitation. Cells were cultured for 48 hours after transfection; then they were replated at low density. Stable transfected cell clones were selected by neomycin G418 (1–0.5 mM).

### 2.2. RT-PCR

Total RNA was isolated from subconfluent cells by the GeneElute Mammalian total RNA Kit (Sigma Chemical Co., St Louis, MO, USA). The cDNA synthesis was performed by the reverse transcriptase ImProm-II starting from random primer examer (Promega, Madison, WI USA). For quantification of LGI1 mRNA, a cDNA segment of (236 bp) was amplified with primers: 5′-TGTAAACTGAAATGGCTAGTGGAA, and 5′-AGTAAAAGGCTGAGCGATGACTAC. Amplification of a BCL2 segment (258 bp) was carried out with primers: 5′-GGTCATGTGTGTGGAGAGCGT, and 5′-ACTTCACTTGTGGCTCAGATAGGC. A BAX fragment (539 bp) was amplified with primers: 5′-CCAGCTCTGAGCAGATCATG and 5′-TCAGCCCATCTTCTTCCAGA. A portion of the Glyceraldehyde-3-phosphate dehydrogenase (GAPDH, 909 bp) was amplified using primers: 5′-GACCCCTTCATTGACCTCAACTACA and 5′-GGTCTTACTCCTTGGAGGCCATGT (Clontech, Palo Alto, CA USA). For the semiquantitative RT-PCR analysis different number of PCR cycles (<25) and serial cDNA dilutions were employed to establish conditions of linear amplification. The PCR products were separated by agarose gel electrophoresis; then the net intensity of ethidium bromide stained bands was determined using the Kodak 1 D Image Analysis Software.

### 2.3. Western Blot

Total cellular proteins (50 *μ*g) were separated on 10%-polyacrylamide gels. The following antibodies (Santa Cruz Biotechnology, Santa Cruz, CA USA) were used to probe blots: antibody C-19 directed to the C-terminal peptide of LGI1 (60 KDa), affinity purified polyclonal antibody (BAX Δ21) against BAX protein (23 KDa), and monoclonal antibodies to Bcl-2 and GAPDH proteins (28 KDa and 36 KDa, respectively. The immunoreactions were revealed by the ECL reagent (Pharmacia Amersham Biotech, Little Chalfont, UK) and quantified by the Kodak 1 D Image Analysis Software by evaluating the net intensity of the bands.

### 2.4. Proliferation Test

An ELISA immunoassay based on BrDU incorporation was employed to measure cell proliferation according to the instruction procedures (Roches Diagnostic, Germany). Cells were plated in quadruplicate on 96 well cluster plates (100 cells/mm^2^) and cultured for 24 hours. Absorbance at (450–690 nm) was measured using a plate reader.

### 2.5. Cell Death

To determine the fraction of dead cells, the lactate dehydrogenase (LDH) released in the medium and that present in intact cells was measured using the CytoTox Non-Radioactive Cytotoxicity Assay (Promega, Madison, WI USA). Cells were plated on 96-well plates (100 cells/mm^2^) and cultured for 24 hours. One half of the culture medium was transferred to a fresh well to determine LDH spontaneously released by dead cells. Lyses of intact cells were performed in the other half of culture medium. Then the conversion of tetrazolium salt (INT) into the red formazan product by LDH activity was measured (Absorbance 490 nm). The fraction of dead cells was calculated as follows: % cytotoxicity = [Spontaneously released LDH × 2 × 100]/Total LDH.

### 2.6. Caspase-3/7

The Apo-ONE Homogeneous caspase-3/7 assay (Promega) was employed to determine caspase-3/7 activity. Cells were seeded on 96-well plates (100 cells/mm^2^), and the caspase-3/7 substrate Z-DEVD-R110 was added 24 hours later. The reaction was carried on for 6 hours, and the measurement of fluorescence was then performed by a plate spectrofluorimeter (485 nm excitation wavelength, 530 nm emission wavelength). LDH assays were performed on replicated cell samples to determine the number of living cells, which was used to normalize caspase activity.

### 2.7. Statistical Analysis

Experimental data were expressed as the mean ± SD. To determine the significance of variations, values were analyzed by Student's *t*-test; levels of *P* < .05 were considered to be significant.

## 3. Results and Discussion

**Figure 1:**
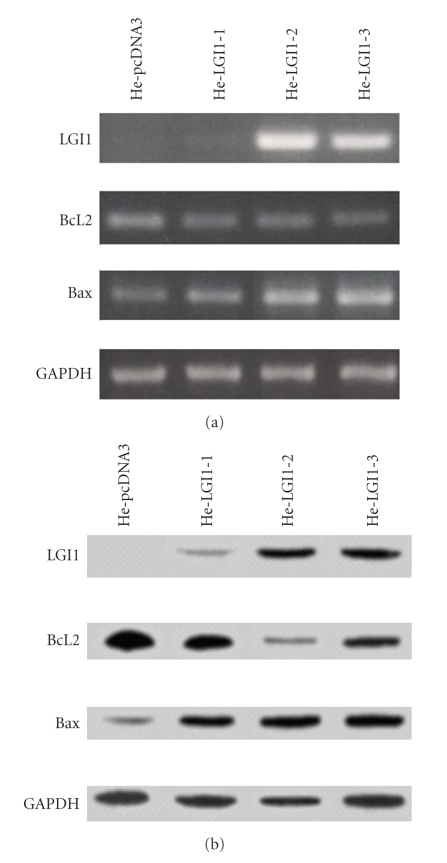
RT-PCR analysis and Western blot. The expression of LGI1, BCL2, BAX, and GAPDH was evaluated in HeLa cell clones expressing LGI1 (HeLGI-1, -2, -3) and control cells transfected with empty vector (He-pcDNA3). The results of RT-PCR analysis are shown in panel (a), those of Western blotting in panel (b).

Stable transfection of HeLa cells with LGI1 cDNA was performed to establish whether expression of LGI1 might affect cell viability. The level of LGI1 expression was evaluated in three cell clones designated He-LGI1-1, He-LGI1-2, and He-LGI1-3, respectively (Figure 1). The results of the quantification of LGI1 mRNA was quite in agreement with the estimated values of LGI1 protein (Table 1). Although LGI1 protein was detected in whole cells sample, the possibility that it might be secreted as it occurred in 293T cells [19] cannot be excluded, since it might be bound to the cell surface.

The endogenous expression of LGI1 in nontransfected HeLa cells and in cell clones stably transfected with empty vector was undetectable by RT-PCR and Western blotting (see He-pcDNA3; Figure 1 and Table 1). The absence of LGI1 expression in human uterine tumors and in normal uterine tissue is supported by the analysis of Expressed Sequence Tags (ESTs) reported by UniGene and is in agreement with lack of LGI1 expression in mouse uterus [20].

**Figure 2:**
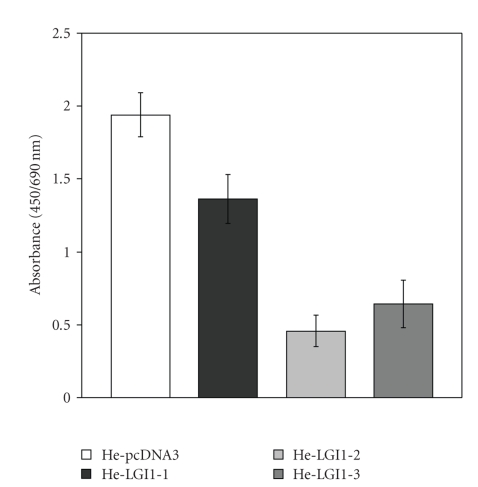
Cell proliferation. Cell clones He-LGI1-1, -2, and -3 and control cells He-pcDNA3 were analyzed by the BrDU incorporation procedure. Proliferation of HeLa cells expressing LGI1 was significantly lower than that of control cells (*n* = 6–11; *P* < .0001).

The BrDU incorporation procedure was employed to evaluate the influence of LGI1 expression on the rate of HeLa cell proliferation. All cell clones expressing LGI1 exhibited a lower proliferation rate in comparison with control cells transfected with empty vector (Figure 2), which were similar to nontransfected HeLa cells (not shown). With He-LGI1-1 cell clone, in which LGI1 expression is very little, decrease of cell proliferation was on average 30%. With He-LGI1-2 cells, expressing the highest LGI1 levels the decrease was 80%, whereas it was 70% with HeLGI1-3 cells, showing intermediate levels of LGI1 expression (Figure 2). The results showed that the inhibitory effect of LGI1 on cell proliferation was proportional with the levels of LGI1 expression.

**Figure 3:**
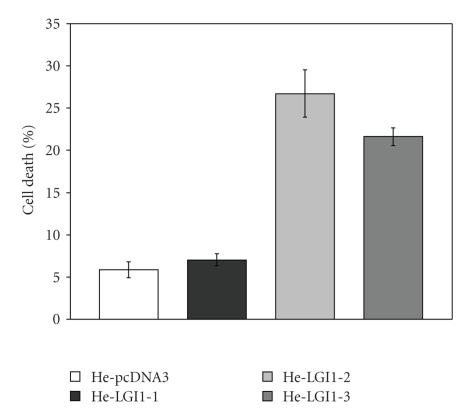
Cell Death. The percentage of cell death was measured on cell clones He-LGI1-1, -2, and -3 and He-pcDNA3 by LDH assays. Cell death was significantly enhanced in He-LGI1-1 in comparison with control cells (*n* = 22–26; *P* = .02), and it was much greater with cell clones He-LGI1-2 and He-LGI1-3 (*n* = 10–26; *P* < .001, 22E-18).

To establish whether expression of LGI1 also might affect survival of HeLa cells we measured the amount of LDH spontaneously released in the medium as a consequence of cell death. The fraction of dead cells was significantly greater in cell clones expressing LGI1 in comparison with control cells (Figure 3). In particular, the percentage of He-LGI1-1 dead cells was on average 7%, slightly but significantly greater than that of He-pcDNA3 cells, which was 5%. The lethal effect was much greater with He-LGI1-2 and He-LGI1-3, whose fractions of dead cells were on average 26% and 21%, respectively. The decline of cell survival was clearly correlated with the levels of LGI1 expression, likewise inhibition of cell proliferation. Since significant effects were obtained with cell clone HeLGI1-1 expressing very low levels of LGI1, it can be suggested that the impairment of cell proliferation and survival was a consequence of the proper LGI1 activity rather than the outcome of artificial overexpression. Furthermore, these effects on cells of epithelial origin exclude the requirement of interactions with proteins specifically expressed in brain for the ability of LGI1 to contrast cell growth and survival.

**Figure 4:**
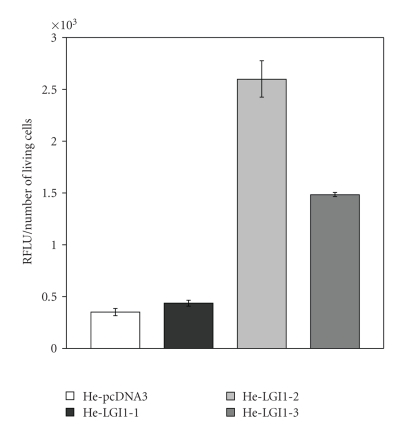
Caspase-3/7 activity. The relative fluorescence (RFLU) emitted upon cleavage of the specific by caspase-3/7 substrate Z-DEVD-R110 was divided to the number of living cells to normalize basal activity. The activity was greater in He-LGI1 cells than in He-pcDNA3 (*n* = 6–8; *P* < .03).

To gain information on the process of HeLa cell death caused by the expression of LGI1 we measured the activity of the apoptosis effectors caspase-3 and -7 in the experimental conditions employed to monitor cell death (Figure 4). The relative fluorescence emitted by the specific caspase-3/7 substrate upon cleavage was particularly enhanced with He-LGI1-2 (7.4-fold) and He-LGI-3 cells (4.2-fold), whereas a modest increase was measured with He-LGI1-1 (Figure 3). The results supported the possibility that expression of LGI1 might trigger apoptosis of HeLa cells.

To substantiate the induction of apoptosis of HeLa cell expressing LGI1 we measured the level of the antiapoptotic BCL2 and of the proapoptotic BAX gene expression, since the relative proportion of these proteins plays a central role in the control of cell survival [18]. A significant decrease of BCL2 mRNA and protein was measured in cell clones expressing LGI1 in comparison with control cells, whereas the expression of BAX was consistently increased (Figure 1; Table 1). In particular, the upregulation of BAX expression was proportional with the levels of LGI1, with a maximum increase of 4.6-fold of BAX protein in He-LGI1-2 (Table 1). The results showed that the antiapoptotic BCL2 gene was downregulated and that of its opponent proapoptotic BAX was upregulated as a consequence of LGI1 expression.

A decreased ratio of BCL2/BAX favors the release of apoptogenic molecules from mitochondria, which activate the intrinsic pathway of apoptosis in various cancer cells, including HeLa [21–26]. Thus LGI1 might increase the susceptibility of HeLa cells to spontaneous apoptosis, similarly to what occurred in neurobalstoma cells [12].

Apparently LGI1 was capable to counteract efficiently the oncogenicity of HPV, specifically of the viral protein E6, which prevents apoptosis by stimulating the ubiquitin-mediated degradation of p53, a transcriptional regulator capable to enhance the expression of BAX and to repress that of BCL2 [16, 27–29]. Thus the balance of these apoptotic factors imposed by E6 to sustain cell survival might be destabilized by LGI1 in favor of apoptosis. Although the mechanism of cell death induced by LGI1 has not been completely elucidated, it seems possible that LGI1 might interfere with central survival pathways such as the PI3K/AKT, as it occurred in neuroblastoma cells [13], because the prosurvival effects of this pathway includes transcriptional regulation of BCL2 and BAX genes [30, 31].

In conclusion, this study supports the possibility that LGI1 might act as tumor suppressor of adenocarcinoma-derived cells, in line with the downregulation of LGI1 reported in Barrett's-related adenocarcinomas and with previous studies on glioblastoma and neuroblastoma cells [9–14].

## Figures and Tables

**Table 1:** Quantification of LGI1, BCL2, and BAX expression in HeLa cell clones. The net intensity values were determined by densitometry and normalized to the average GAPDH content of each cell sample. The values of BCL2 and BAX are shown as ratio with He-pcDNA3 values. The expression of BCL2 and BAX in all cell clones expressing LGI1 significantly differs from that of pc-DNA3 cells (*n* = 3 − 6; *P* < .01).

Cell clones	LGI1		BCL2		BAX	
	mRNA	Protein	mRNA	Protein	mRNA	Protein

He-pcDNA3	0	0	1	1	1	1
He-LGI1-1	0.11 ± 0.02	0.30 ± 0.01	0.67 ± 0.07	0.72 ± 0.09	1.50 ± 0.14	3.39 ± 0.10
He-LGI1-2	2.61 ± 0.14	1.48 ± 0.26	0.52 ± 0.02	0.56 ± 0.09	1.75 ± 0.07	4.67 ± 0.59
He-LGI1-3	1.89 ± 0.35	1.22 ± 0.07	0.70 ± 0.03	0.86 ± 0.06	1.31 ± 0.02	2.27 ± 0.13
